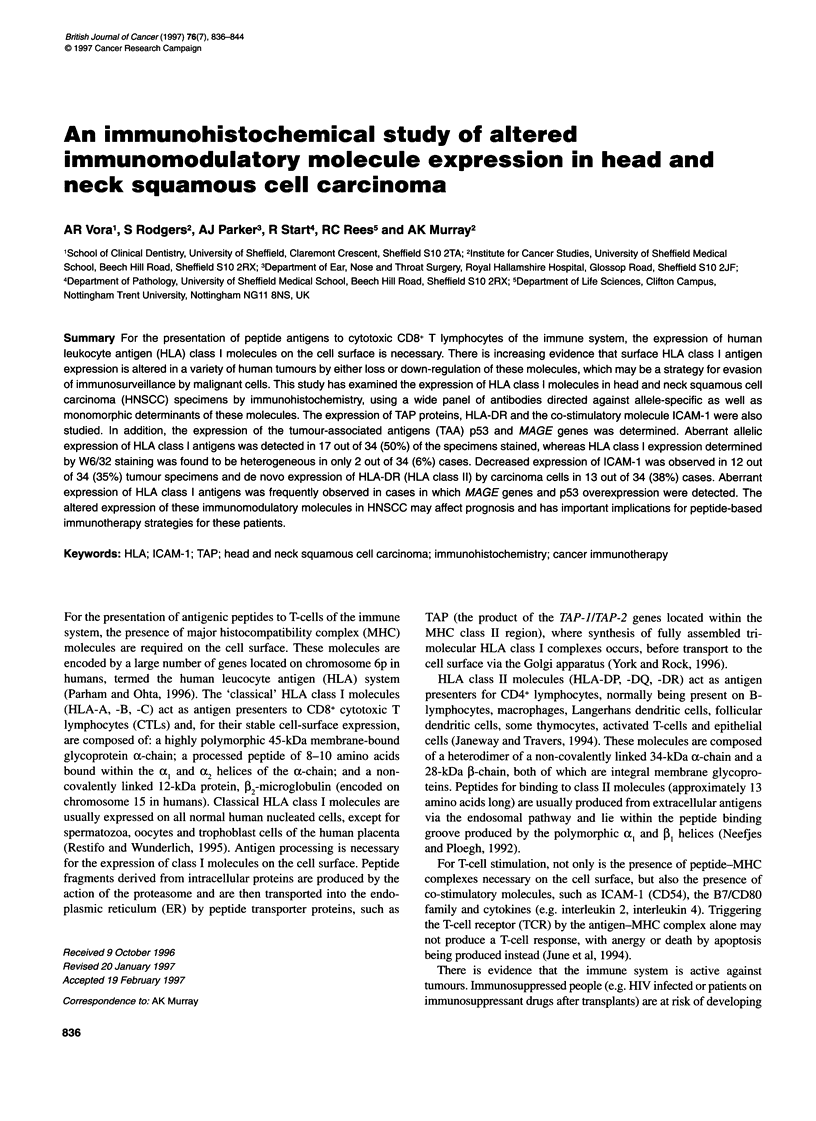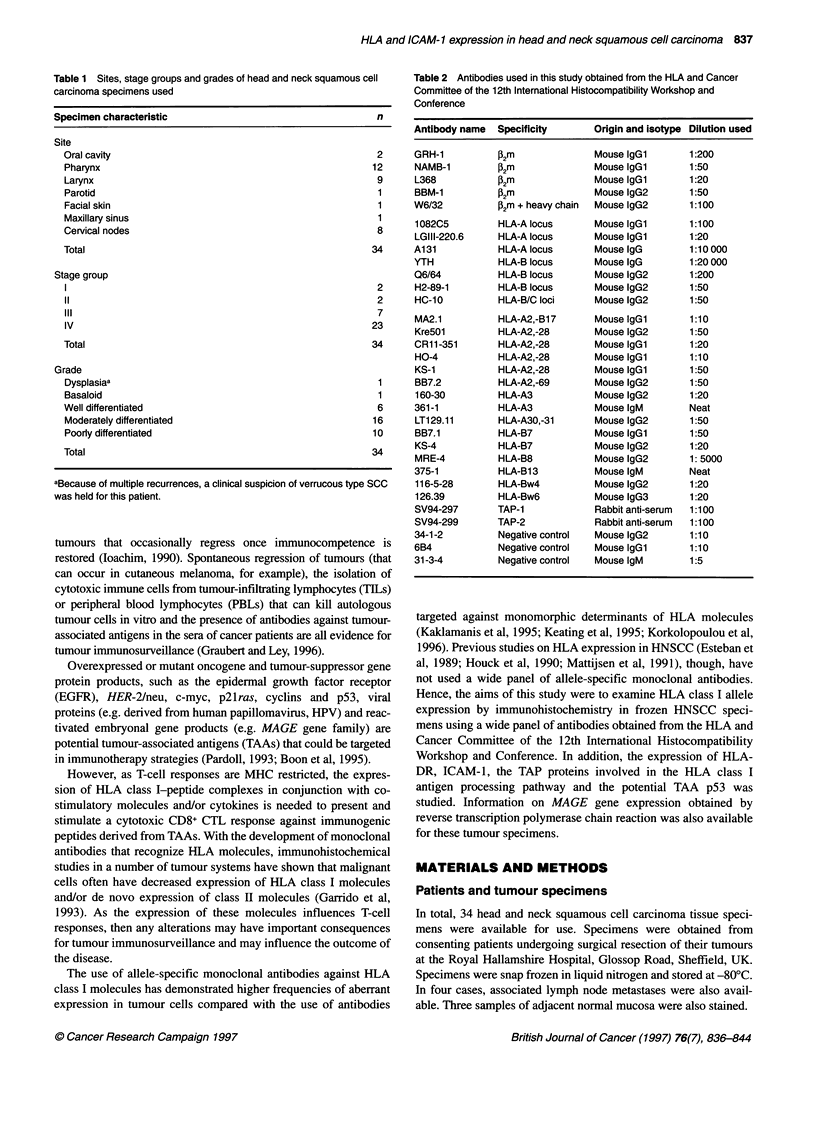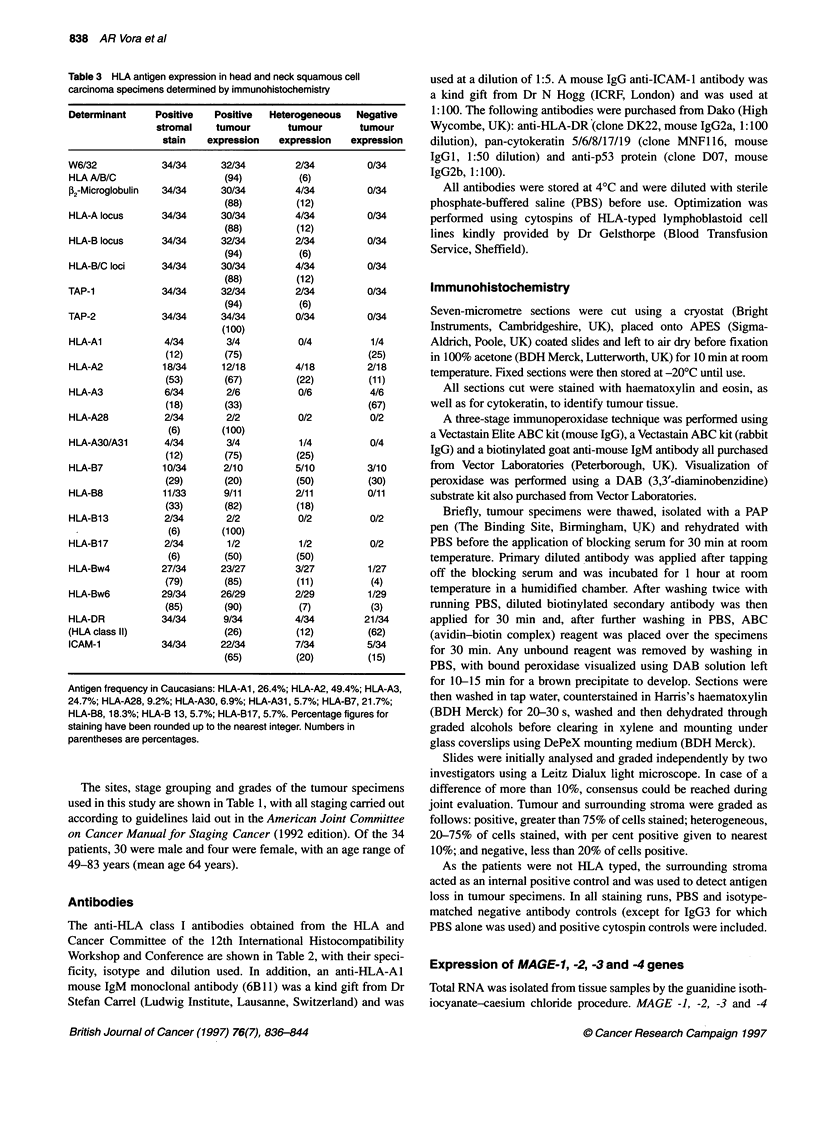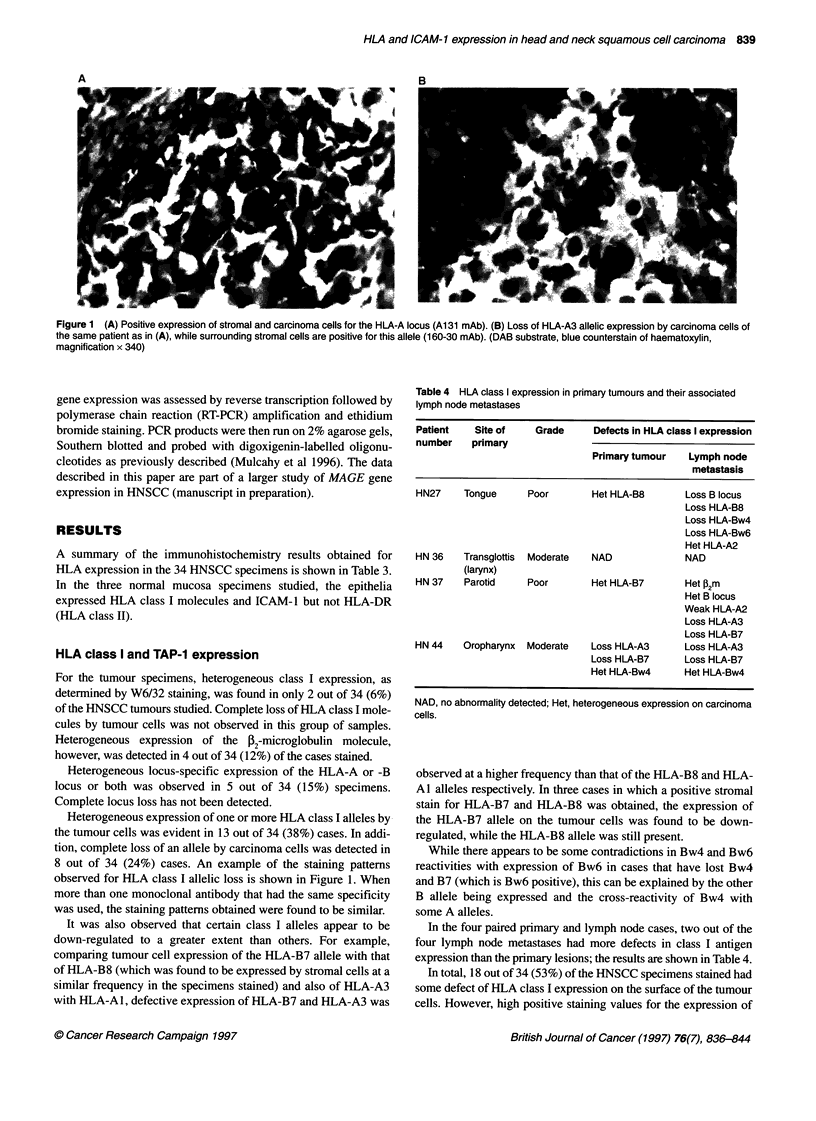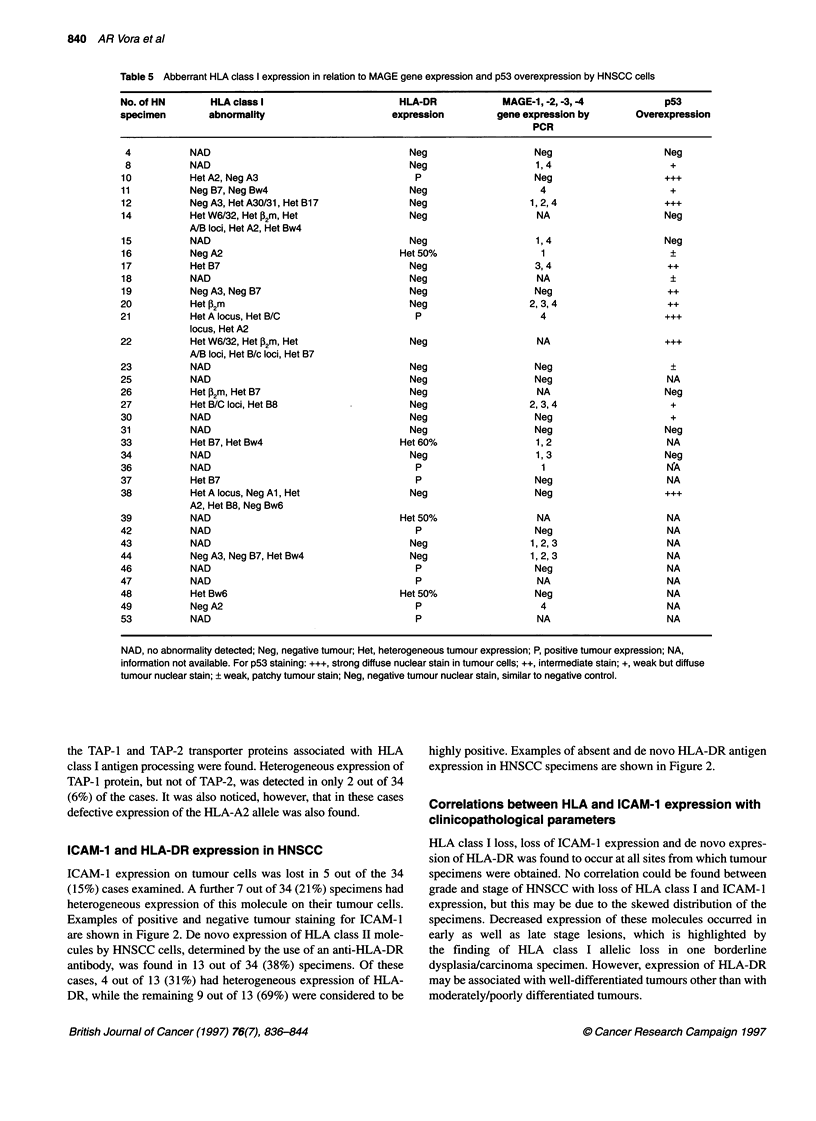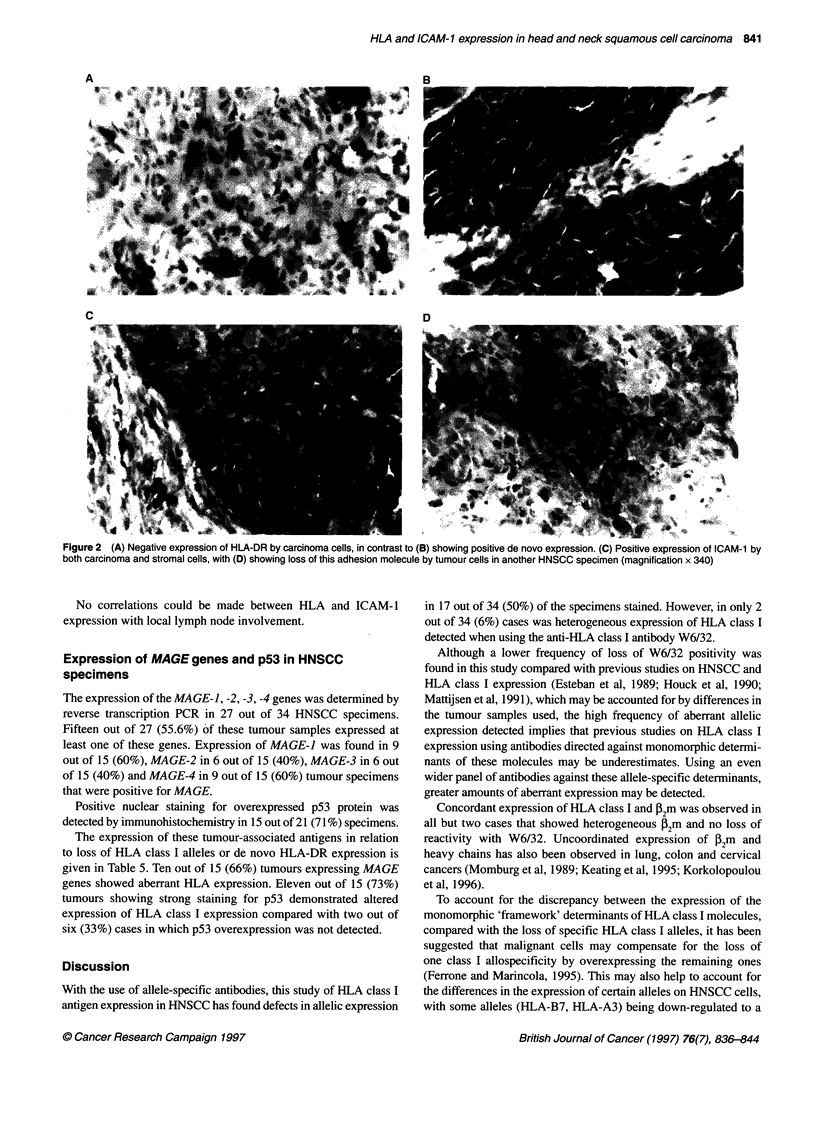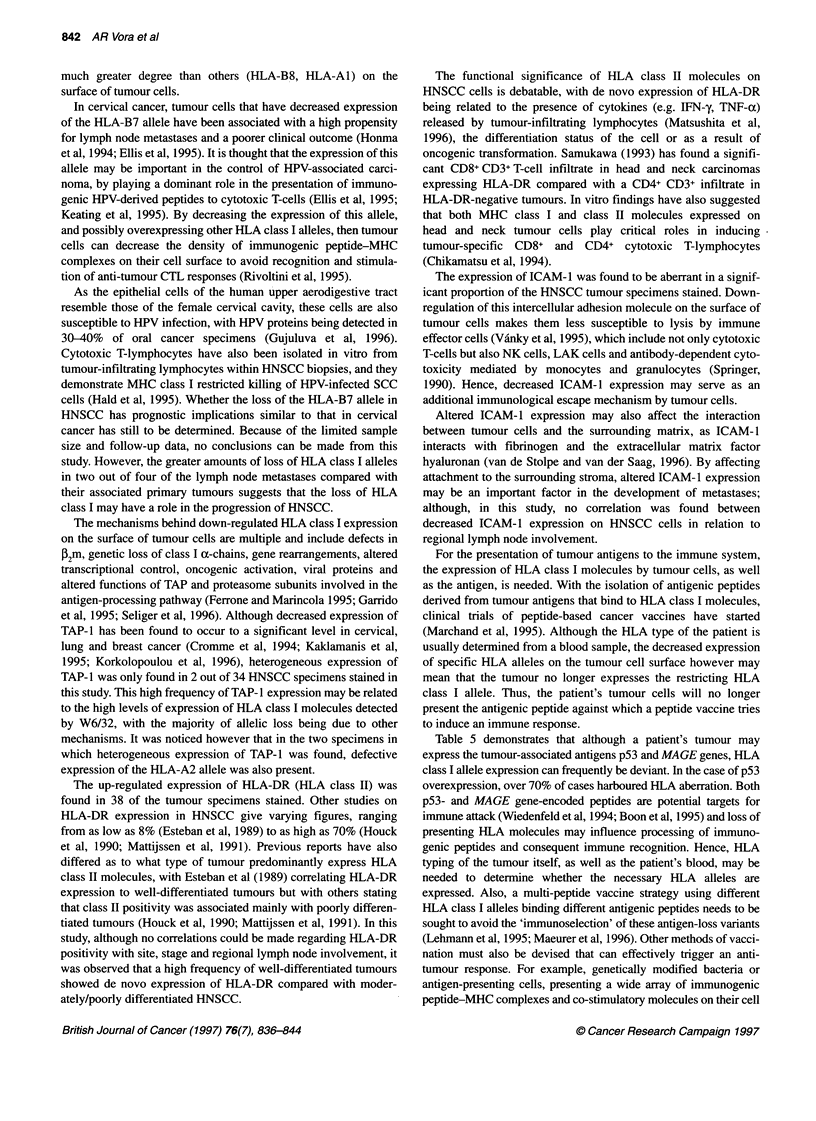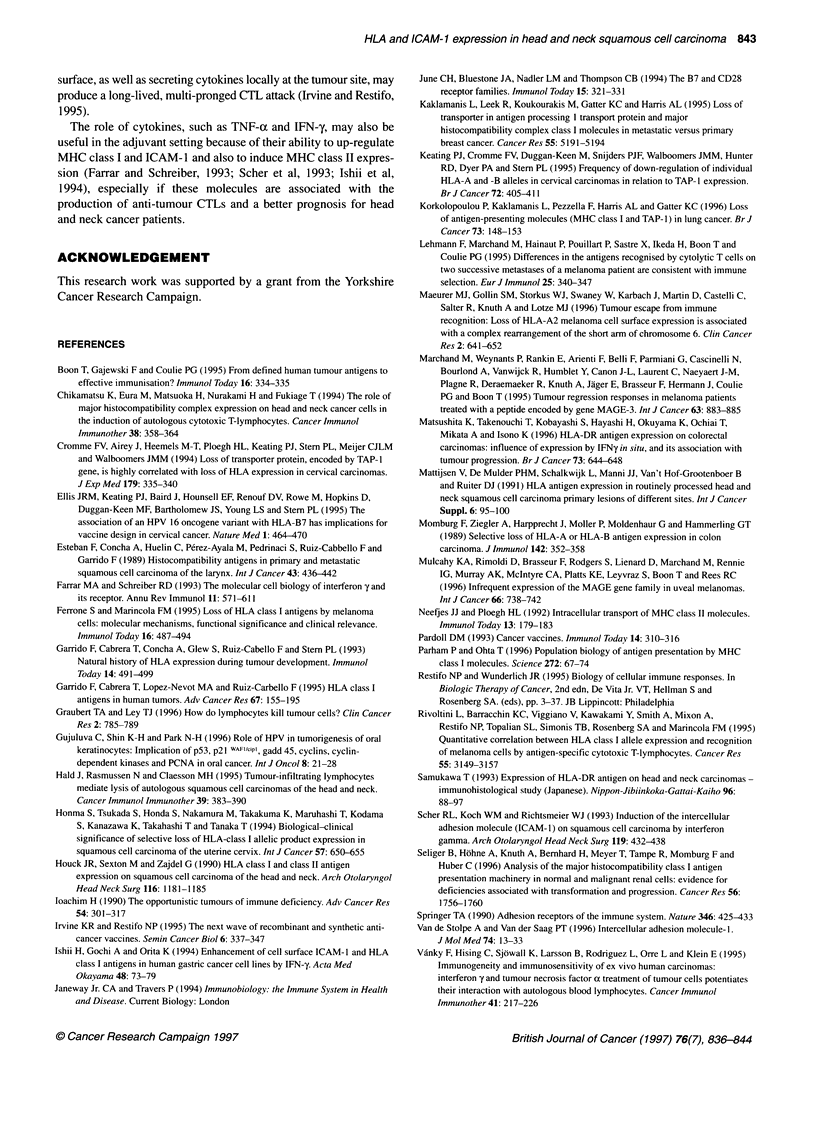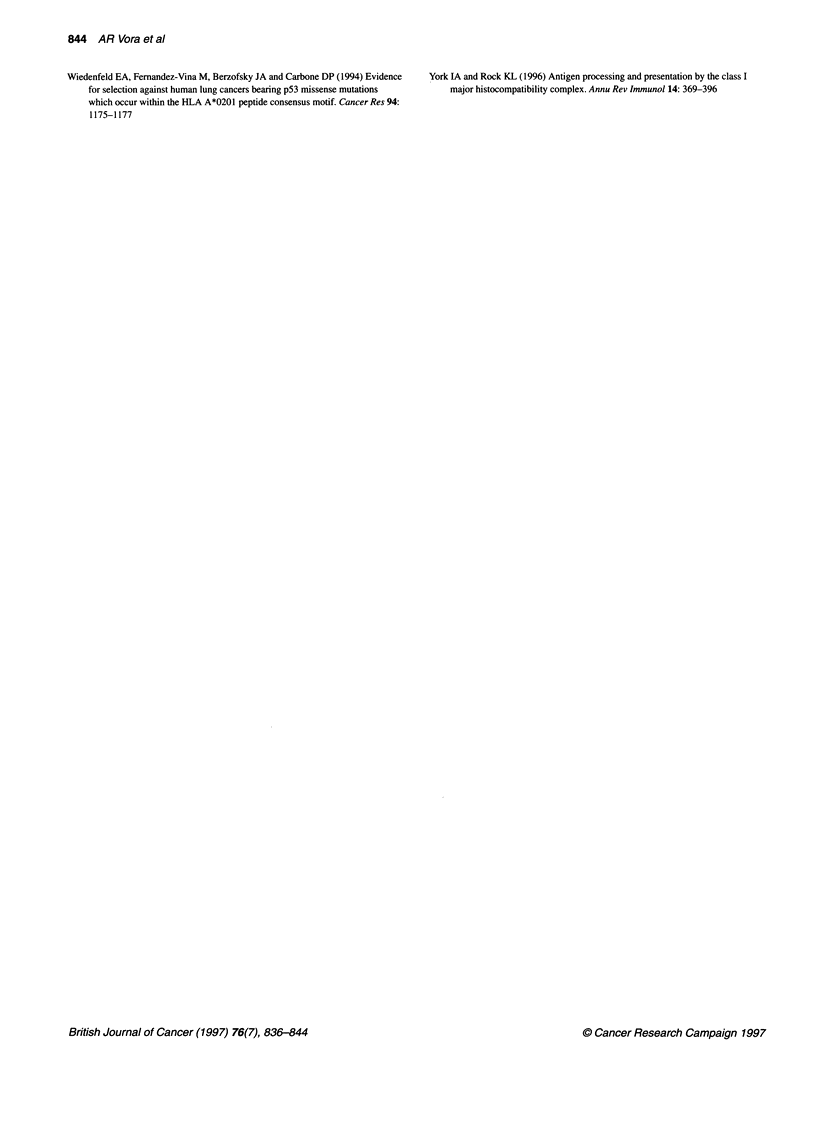# An immunohistochemical study of altered immunomodulatory molecule expression in head and neck squamous cell carcinoma.

**DOI:** 10.1038/bjc.1997.472

**Published:** 1997

**Authors:** A. R. Vora, S. Rodgers, A. J. Parker, R. Start, R. C. Rees, A. K. Murray

**Affiliations:** School of Clinical Dentistry, University of Sheffield, UK.

## Abstract

**Images:**


British Journal of Cancer (1997) 76(7), 836–844
© 1997 Cancer Research Campaign

### An immunohistochemical study of altered immunomodulatory molecule expression in head and neck squamous cell carcinoma

**AR Vora[1], S Rodgers[2], AJ Parker[3], R Start[4], RC Rees[5] and AK Murray[2]**

[1]School of Clinical Dentistry, University of Sheffield, Claremont Crescent, Sheffield S10 2TA; [2]Institute for Cancer Studies, University of Sheffield Medical School, Beech Hill Road, Sheffield S10 2RX; [3]Department of Ear, Nose and Throat Surgery, Royal Hallamshire Hospital, Glossop Road, Sheffield S10 2JF; [4]Department of Pathology, University of Sheffield Medical School, Beech Hill Road, Sheffield S10 2RX; [5]Department of Life Sciences, Clifton Campus, Nottingham Trent University, Nottingham NG11 8NS, UK

**Summary** For the presentation of peptide antigens to cytotoxic CD8+ T lymphocytes of the immune system, the expression of human leukocyte antigen (HLA) class I molecules on the cell surface is necessary. There is increasing evidence that surface HLA class I antigen expression is altered in a variety of human tumours by either loss or down-regulation of these molecules, which may be a strategy for evasion of immunosurveillance by malignant cells. This study has examined the expression of HLA class I molecules in head and neck squamous cell carcinoma (HNSCC) specimens by immunohistochemistry, using a wide panel of antibodies directed against allele-specific as well as monomorphic determinants of these molecules. The expression of TAP proteins, HLA-DR and the co-stimulatory molecule ICAM-1 were also studied. In addition, the expression of the tumour-associated antigens (TAA) p53 and *MAGE* genes was determined. Aberrant allelic expression of HLA class I antigens was detected in 17 out of 34 (50%) of the specimens stained, whereas HLA class I expression determined by W6/32 staining was found to be heterogeneous in only 2 out of 34 (6%) cases. Decreased expression of ICAM-1 was observed in 12 out of 34 (35%) tumour specimens and de novo expression of HLA-DR (HLA class II) by carcinoma cells in 13 out of 34 (38%) cases. Aberrant expression of HLA class I antigens was frequently observed in cases in which *MAGE* genes and p53 overexpression were detected. The altered expression of these immunomodulatory molecules in HNSCC may affect prognosis and has important implications for peptide-based immunotherapy strategies for these patients.

**Keywords:** HLA; ICAM-1; TAP; head and neck squamous cell carcinoma; immunohistochemistry; cancer immunotherapy

For the presentation of antigenic peptides to T-cells of the immune system, the presence of major histocompatibility complex (MHC) molecules are required on the cell surface. These molecules are encoded by a large number of genes located on chromosome 6p in humans, termed the human leucocyte antigen (HLA) system (Parham and Ohta, 1996). The 'classical' HLA class I molecules (HLA-A, -B, -C) act as antigen presenters to CD8+ cytotoxic T lymphocytes (CTLs) and, for their stable cell-surface expression, are composed of: a highly polymorphic 45-kDa membrane-bound glycoprotein α-chain; a processed peptide of 8–10 amino acids bound within the $\alpha_1$ and $\alpha_2$ helices of the α-chain; and a non-covalently linked 12-kDa protein, $\beta_2$-microglobulin (encoded on chromosome 15 in humans). Classical HLA class I molecules are usually expressed on all normal human nucleated cells, except for spermatozoa, oocytes and trophoblast cells of the human placenta (Restifo and Wunderlich, 1995). Antigen processing is necessary for the expression of class I molecules on the cell surface. Peptide fragments derived from intracellular proteins are produced by the action of the proteasome and are then transported into the endoplasmic reticulum (ER) by peptide transporter proteins, such as TAP (the product of the *TAP-1*/*TAP-2* genes located within the MHC class II region), where synthesis of fully assembled trimolecular HLA class I complexes occurs, before transport to the cell surface via the Golgi apparatus (York and Rock, 1996).

*Received 9 October 1996*
*Revised 20 January 1997*
*Accepted 19 February 1997*

*Correspondence to:* AK Murray

HLA class II molecules (HLA-DP, -DQ, -DR) act as antigen presenters for CD4+ lymphocytes, normally being present on B-lymphocytes, macrophages, Langerhans dendritic cells, follicular dendritic cells, some thymocytes, activated T-cells and epithelial cells (Janeway and Travers, 1994). These molecules are composed of a heterodimer of a non-covalently linked 34-kDa α-chain and a 28-kDa β-chain, both of which are integral membrane glycoproteins. Peptides for binding to class II molecules (approximately 13 amino acids long) are usually produced from extracellular antigens via the endosomal pathway and lie within the peptide binding groove produced by the polymorphic $\alpha_1$ and $\beta_1$ helices (Neefjes and Ploegh, 1992).

For T-cell stimulation, not only is the presence of peptide–MHC complexes necessary on the cell surface, but also the presence of co-stimulatory molecules, such as ICAM-1 (CD54), the B7/CD80 family and cytokines (e.g. interleukin 2, interleukin 4). Triggering the T-cell receptor (TCR) by the antigen–MHC complex alone may not produce a T-cell response, with anergy or death by apoptosis being produced instead (June et al, 1994).

There is evidence that the immune system is active against tumours. Immunosuppressed people (e.g. HIV infected or patients on immunosuppressant drugs after transplants) are at risk of developing

**Table 1** Sites, stage groups and grades of head and neck squamous cell carcinoma specimens used

| Specimen characteristic | n |
|---|---|
| Site | |
| Oral cavity | 2 |
| Pharynx | 12 |
| Larynx | 9 |
| Parotid | 1 |
| Facial skin | 1 |
| Maxillary sinus | 1 |
| Cervical nodes | 8 |
| Total | 34 |
| Stage group | |
| I | 2 |
| II | 2 |
| III | 7 |
| IV | 23 |
| Total | 34 |
| Grade | |
| Dysplasia[a] | 1 |
| Basaloid | 1 |
| Well differentiated | 6 |
| Moderately differentiated | 16 |
| Poorly differentiated | 10 |
| Total | 34 |

[a]Because of multiple recurrences, a clinical suspicion of verrucous type SCC was held for this patient.

**Table 2** Antibodies used in this study obtained from the HLA and Cancer Committee of the 12th International Histocompatibility Workshop and Conference

| Antibody name | Specificity | Origin and isotype | Dilution used |
|---|---|---|---|
| GRH-1 | $\beta_2$m | Mouse IgG1 | 1:200 |
| NAMB-1 | $\beta_2$m | Mouse IgG1 | 1:50 |
| L368 | $\beta_2$m | Mouse IgG1 | 1:20 |
| BBM-1 | $\beta_2$m | Mouse IgG2 | 1:50 |
| W6/32 | $\beta_2$m + heavy chain | Mouse IgG2 | 1:100 |
| 1082C5 | HLA-A locus | Mouse IgG1 | 1:100 |
| LGIII-220.6 | HLA-A locus | Mouse IgG1 | 1:20 |
| A131 | HLA-A locus | Mouse IgG | 1:10 000 |
| YTH | HLA-B locus | Mouse IgG | 1:20 000 |
| Q6/64 | HLA-B locus | Mouse IgG2 | 1:200 |
| H2-89-1 | HLA-B locus | Mouse IgG2 | 1:50 |
| HC-10 | HLA-B/C loci | Mouse IgG2 | 1:50 |
| MA2.1 | HLA-A2,-B17 | Mouse IgG1 | 1:10 |
| Kre501 | HLA-A2,-28 | Mouse IgG2 | 1:50 |
| CR11-351 | HLA-A2,-28 | Mouse IgG1 | 1:20 |
| HO-4 | HLA-A2,-28 | Mouse IgG1 | 1:10 |
| KS-1 | HLA-A2,-28 | Mouse IgG1 | 1:50 |
| BB7.2 | HLA-A2,-69 | Mouse IgG2 | 1:50 |
| 160-30 | HLA-A3 | Mouse IgG2 | 1:20 |
| 361-1 | HLA-A3 | Mouse IgM | Neat |
| LT129.11 | HLA-A30,-31 | Mouse IgG2 | 1:50 |
| BB7.1 | HLA-B7 | Mouse IgG1 | 1:50 |
| KS-4 | HLA-B7 | Mouse IgG2 | 1:20 |
| MRE-4 | HLA-B8 | Mouse IgG2 | 1: 5000 |
| 375-1 | HLA-B13 | Mouse IgM | Neat |
| 116-5-28 | HLA-Bw4 | Mouse IgG2 | 1:20 |
| 126.39 | HLA-Bw6 | Mouse IgG3 | 1:20 |
| SV94-297 | TAP-1 | Rabbit anti-serum | 1:100 |
| SV94-299 | TAP-2 | Rabbit anti-serum | 1:100 |
| 34-1-2 | Negative control | Mouse IgG2 | 1:10 |
| 6B4 | Negative control | Mouse IgG1 | 1:10 |
| 31-3-4 | Negative control | Mouse IgM | 1:5 |

tumours that occasionally regress once immunocompetence is restored (Ioachim, 1990). Spontaneous regression of tumours (that can occur in cutaneous melanoma, for example), the isolation of cytotoxic immune cells from tumour-infiltrating lymphocytes (TILs) or peripheral blood lymphocytes (PBLs) that can kill autologous tumour cells in vitro and the presence of antibodies against tumour-associated antigens in the sera of cancer patients are all evidence for tumour immunosurveillance (Graubert and Ley, 1996).

Overexpressed or mutant oncogene and tumour-suppressor gene protein products, such as the epidermal growth factor receptor (EGFR), *HER*-2/neu, c-myc, p21*ras*, cyclins and p53, viral proteins (e.g. derived from human papillomavirus, HPV) and reactivated embryonal gene products (e.g. *MAGE* gene family) are potential tumour-associated antigens (TAAs) that could be targeted in immunotherapy strategies (Pardoll, 1993; Boon et al, 1995).

However, as T-cell responses are MHC restricted, the expression of HLA class I–peptide complexes in conjunction with co-stimulatory molecules and/or cytokines is needed to present and stimulate a cytotoxic $CD8^+$ CTL response against immunogenic peptides derived from TAAs. With the development of monoclonal antibodies that recognize HLA molecules, immunohistochemical studies in a number of tumour systems have shown that malignant cells often have decreased expression of HLA class I molecules and/or de novo expression of class II molecules (Garrido et al, 1993). As the expression of these molecules influences T-cell responses, then any alterations may have important consequences for tumour immunosurveillance and may influence the outcome of the disease.

The use of allele-specific monoclonal antibodies against HLA class I molecules has demonstrated higher frequencies of aberrant expression in tumour cells compared with the use of antibodies targeted against monomorphic determinants of HLA molecules (Kaklamanis et al, 1995; Keating et al, 1995; Korkolopoulou et al, 1996). Previous studies on HLA expression in HNSCC (Esteban et al, 1989; Houck et al, 1990; Mattijsen et al, 1991), though, have not used a wide panel of allele-specific monoclonal antibodies. Hence, the aims of this study were to examine HLA class I allele expression by immunohistochemistry in frozen HNSCC specimens using a wide panel of antibodies obtained from the HLA and Cancer Committee of the 12th International Histocompatibility Workshop and Conference. In addition, the expression of HLA-DR, ICAM-1, the TAP proteins involved in the HLA class I antigen processing pathway and the potential TAA p53 was studied. Information on *MAGE* gene expression obtained by reverse transcription polymerase chain reaction was also available for these tumour specimens.

### MATERIALS AND METHODS

### Patients and tumour specimens

In total, 34 head and neck squamous cell carcinoma tissue specimens were available for use. Specimens were obtained from consenting patients undergoing surgical resection of their tumours at the Royal Hallamshire Hospital, Glossop Road, Sheffield, UK. Specimens were snap frozen in liquid nitrogen and stored at –80°C. In four cases, associated lymph node metastases were also available. Three samples of adjacent normal mucosa were also stained.

© Cancer Research Campaign 1997

**Table 3** HLA antigen expression in head and neck squamous cell carcinoma specimens determined by immunohistochemistry

| Determinant | Positive stromal stain | Positive tumour expression | Heterogeneous tumour expression | Negative tumour expression |
|---|---|---|---|---|
| W6/32 HLA A/B/C | 34/34 | 32/34 (94) | 2/34 (6) | 0/34 |
| $\beta_2$-Microglobulin | 34/34 | 30/34 (88) | 4/34 (12) | 0/34 |
| HLA-A locus | 34/34 | 30/34 (88) | 4/34 (12) | 0/34 |
| HLA-B locus | 34/34 | 32/34 (94) | 2/34 (6) | 0/34 |
| HLA-B/C loci | 34/34 | 30/34 (88) | 4/34 (12) | 0/34 |
| TAP-1 | 34/34 | 32/34 (94) | 2/34 (6) | 0/34 |
| TAP-2 | 34/34 | 34/34 (100) | 0/34 | 0/34 |
| HLA-A1 | 4/34 (12) | 3/4 (75) | 0/4 | 1/4 (25) |
| HLA-A2 | 18/34 (53) | 12/18 (67) | 4/18 (22) | 2/18 (11) |
| HLA-A3 | 6/34 (18) | 2/6 (33) | 0/6 | 4/6 (67) |
| HLA-A28 | 2/34 (6) | 2/2 (100) | 0/2 | 0/2 |
| HLA-A30/A31 | 4/34 (12) | 3/4 (75) | 1/4 (25) | 0/4 |
| HLA-B7 | 10/34 (29) | 2/10 (20) | 5/10 (50) | 3/10 (30) |
| HLA-B8 | 11/33 (33) | 9/11 (82) | 2/11 (18) | 0/11 |
| HLA-B13 | 2/34 (6) | 2/2 (100) | 0/2 | 0/2 |
| HLA-B17 | 2/34 (6) | 1/2 (50) | 1/2 (50) | 0/2 |
| HLA-Bw4 | 27/34 (79) | 23/27 (85) | 3/27 (11) | 1/27 (4) |
| HLA-Bw6 | 29/34 (85) | 26/29 (90) | 2/29 (7) | 1/29 (3) |
| HLA-DR (HLA class II) | 34/34 | 9/34 (26) | 4/34 (12) | 21/34 (62) |
| ICAM-1 | 34/34 | 22/34 (65) | 7/34 (20) | 5/34 (15) |

Antigen frequency in Caucasians: HLA-A1, 26.4%; HLA-A2, 49.4%; HLA-A3, 24.7%; HLA-A28, 9.2%; HLA-A30, 6.9%; HLA-A31, 5.7%; HLA-B7, 21.7%; HLA-B8, 18.3%; HLA-B 13, 5.7%; HLA-B17, 5.7%. Percentage figures for staining have been rounded up to the nearest integer. Numbers in parentheses are percentages.

The sites, stage grouping and grades of the tumour specimens used in this study are shown in Table 1, with all staging carried out according to guidelines laid out in the *American Joint Committee on Cancer Manual for Staging Cancer* (1992 edition). Of the 34 patients, 30 were male and four were female, with an age range of 49–83 years (mean age 64 years).

### Antibodies

The anti-HLA class I antibodies obtained from the HLA and Cancer Committee of the 12th International Histocompatibility Workshop and Conference are shown in Table 2, with their specificity, isotype and dilution used. In addition, an anti-HLA-A1 mouse IgM monoclonal antibody (6B11) was a kind gift from Dr Stefan Carrel (Ludwig Institute, Lausanne, Switzerland) and was used at a dilution of 1:5. A mouse IgG anti-ICAM-1 antibody was a kind gift from Dr N Hogg (ICRF, London) and was used at 1:100. The following antibodies were purchased from Dako (High Wycombe, UK): anti-HLA-DR (clone DK22, mouse IgG2a, 1:100 dilution), pan-cytokeratin 5/6/8/17/19 (clone MNF116, mouse IgG1, 1:50 dilution) and anti-p53 protein (clone D07, mouse IgG2b, 1:100).

All antibodies were stored at 4°C and were diluted with sterile phosphate-buffered saline (PBS) before use. Optimization was performed using cytospins of HLA-typed lymphoblastoid cell lines kindly provided by Dr Gelsthorpe (Blood Transfusion Service, Sheffield).

### Immunohistochemistry

Seven-micrometre sections were cut using a cryostat (Bright Instruments, Cambridgeshire, UK), placed onto APES (Sigma-Aldrich, Poole, UK) coated slides and left to air dry before fixation in 100% acetone (BDH Merck, Lutterworth, UK) for 10 min at room temperature. Fixed sections were then stored at –20°C until use.

All sections cut were stained with haematoxylin and eosin, as well as for cytokeratin, to identify tumour tissue.

A three-stage immunoperoxidase technique was performed using a Vectastain Elite ABC kit (mouse IgG), a Vectastain ABC kit (rabbit IgG) and a biotinylated goat anti-mouse IgM antibody all purchased from Vector Laboratories (Peterborough, UK). Visualization of peroxidase was performed using a DAB (3,3′-diaminobenzidine) substrate kit also purchased from Vector Laboratories.

Briefly, tumour specimens were thawed, isolated with a PAP pen (The Binding Site, Birmingham, UK) and rehydrated with PBS before the application of blocking serum for 30 min at room temperature. Primary diluted antibody was applied after tapping off the blocking serum and was incubated for 1 hour at room temperature in a humidified chamber. After washing twice with running PBS, diluted biotinylated secondary antibody was then applied for 30 min and, after further washing in PBS, ABC (avidin–biotin complex) reagent was placed over the specimens for 30 min. Any unbound reagent was removed by washing in PBS, with bound peroxidase visualized using DAB solution left for 10–15 min for a brown precipitate to develop. Sections were then washed in tap water, counterstained in Harris's haematoxylin (BDH Merck) for 20–30 s, washed and then dehydrated through graded alcohols before clearing in xylene and mounting under glass coverslips using DePeX mounting medium (BDH Merck).

Slides were initially analysed and graded independently by two investigators using a Leitz Dialux light microscope. In case of a difference of more than 10%, consensus could be reached during joint evaluation. Tumour and surrounding stroma were graded as follows: positive, greater than 75% of cells stained; heterogeneous, 20–75% of cells stained, with per cent positive given to nearest 10%; and negative, less than 20% of cells positive.

As the patients were not HLA typed, the surrounding stroma acted as an internal positive control and was used to detect antigen loss in tumour specimens. In all staining runs, PBS and isotype-matched negative antibody controls (except for IgG3 for which PBS alone was used) and positive cytospin controls were included.

### Expression of *MAGE-1*, *-2*, *-3* and *-4* genes

Total RNA was isolated from tissue samples by the guanidine isothiocyanate–caesium chloride procedure. *MAGE -1, -2, -3* and *-4*

© Cancer Research Campaign 1997

**Figure 1** (**A**) Positive expression of stromal and carcinoma cells for the HLA-A locus (A131 mAb). (**B**) Loss of HLA-A3 allelic expression by carcinoma cells of the same patient as in (**A**), while surrounding stromal cells are positive for this allele (160-30 mAb). (DAB substrate, blue counterstain of haematoxylin, magnification × 340)

gene expression was assessed by reverse transcription followed by polymerase chain reaction (RT-PCR) amplification and ethidium bromide staining. PCR products were then run on 2% agarose gels, Southern blotted and probed with digoxigenin-labelled oligonucleotides as previously described (Mulcahy et al 1996). The data described in this paper are part of a larger study of *MAGE* gene expression in HNSCC (manuscript in preparation).

### RESULTS

A summary of the immunohistochemistry results obtained for HLA expression in the 34 HNSCC specimens is shown in Table 3. In the three normal mucosa specimens studied, the epithelia expressed HLA class I molecules and ICAM-1 but not HLA-DR (HLA class II).

### HLA class I and TAP-1 expression

For the tumour specimens, heterogeneous class I expression, as determined by W6/32 staining, was found in only 2 out of 34 (6%) of the HNSCC tumours studied. Complete loss of HLA class I molecules by tumour cells was not observed in this group of samples. Heterogeneous expression of the $\beta_2$-microglobulin molecule, however, was detected in 4 out of 34 (12%) of the cases stained.

Heterogeneous locus-specific expression of the HLA-A or -B locus or both was observed in 5 out of 34 (15%) specimens. Complete locus loss has not been detected.

Heterogeneous expression of one or more HLA class I alleles by the tumour cells was evident in 13 out of 34 (38%) cases. In addition, complete loss of an allele by carcinoma cells was detected in 8 out of 34 (24%) cases. An example of the staining patterns observed for HLA class I allelic loss is shown in Figure 1. When more than one monoclonal antibody that had the same specificity was used, the staining patterns obtained were found to be similar.

It was also observed that certain class I alleles appear to be down-regulated to a greater extent than others. For example, comparing tumour cell expression of the HLA-B7 allele with that of HLA-B8 (which was found to be expressed by stromal cells at a similar frequency in the specimens stained) and also of HLA-A3 with HLA-A1, defective expression of HLA-B7 and HLA-A3 was observed at a higher frequency than that of the HLA-B8 and HLA-A1 alleles respectively. In three cases in which a positive stromal stain for HLA-B7 and HLA-B8 was obtained, the expression of the HLA-B7 allele on the tumour cells was found to be down-regulated, while the HLA-B8 allele was still present.

While there appears to be some contradictions in Bw4 and Bw6 reactivities with expression of Bw6 in cases that have lost Bw4 and B7 (which is Bw6 positive), this can be explained by the other B allele being expressed and the cross-reactivity of Bw4 with some A alleles.

In the four paired primary and lymph node cases, two out of the four lymph node metastases had more defects in class I antigen expression than the primary lesions; the results are shown in Table 4.

In total, 18 out of 34 (53%) of the HNSCC specimens stained had some defect of HLA class I expression on the surface of the tumour cells. However, high positive staining values for the expression of

**Table 4** HLA class I expression in primary tumours and their associated lymph node metastases

| Patient number | Site of primary | Grade | Defects in HLA class I expression | |
|---|---|---|---|---|
| | | | Primary tumour | Lymph node metastasis |
| HN27 | Tongue | Poor | Het HLA-B8 | Loss B locus<br>Loss HLA-B8<br>Loss HLA-Bw4<br>Loss HLA-Bw6<br>Het HLA-A2 |
| HN 36 | Transglottis (larynx) | Moderate | NAD | NAD |
| HN 37 | Parotid | Poor | Het HLA-B7 | Het $\beta_2$m<br>Het B locus<br>Weak HLA-A2<br>Loss HLA-A3<br>Loss HLA-B7 |
| HN 44 | Oropharynx | Moderate | Loss HLA-A3<br>Loss HLA-B7<br>Het HLA-Bw4 | Loss HLA-A3<br>Loss HLA-B7<br>Het HLA-Bw4 |

NAD, no abnormality detected; Het, heterogeneous expression on carcinoma cells.

© Cancer Research Campaign 1997

**Table 5** Abberrant HLA class I expression in relation to MAGE gene expression and p53 overexpression by HNSCC cells

| No. of HN specimen | HLA class I abnormality | HLA-DR expression | MAGE-1, -2, -3, -4 gene expression by PCR | p53 Overexpression |
|---|---|---|---|---|
| 4 | NAD | Neg | Neg | Neg |
| 8 | NAD | Neg | 1, 4 | + |
| 10 | Het A2, Neg A3 | P | Neg | +++ |
| 11 | Neg B7, Neg Bw4 | Neg | 4 | + |
| 12 | Neg A3, Het A30/31, Het B17 | Neg | 1, 2, 4 | +++ |
| 14 | Het W6/32, Het $\beta_2$m, Het A/B loci, Het A2, Het Bw4 | Neg | NA | Neg |
| 15 | NAD | Neg | 1, 4 | Neg |
| 16 | Neg A2 | Het 50% | 1 | ± |
| 17 | Het B7 | Neg | 3, 4 | ++ |
| 18 | NAD | Neg | NA | ± |
| 19 | Neg A3, Neg B7 | Neg | Neg | ++ |
| 20 | Het $\beta_2$m | Neg | 2, 3, 4 | ++ |
| 21 | Het A locus, Het B/C locus, Het A2 | P | 4 | +++ |
| 22 | Het W6/32, Het $\beta_2$m, Het A/B loci, Het B/c loci, Het B7 | Neg | NA | +++ |
| 23 | NAD | Neg | Neg | ± |
| 25 | NAD | Neg | Neg | NA |
| 26 | Het $\beta_2$m, Het B7 | Neg | NA | Neg |
| 27 | Het B/C loci, Het B8 | Neg | 2, 3, 4 | + |
| 30 | NAD | Neg | Neg | + |
| 31 | NAD | Neg | Neg | Neg |
| 33 | Het B7, Het Bw4 | Het 60% | 1, 2 | NA |
| 34 | NAD | Neg | 1, 3 | Neg |
| 36 | NAD | P | 1 | NA |
| 37 | Het B7 | P | Neg | NA |
| 38 | Het A locus, Neg A1, Het A2, Het B8, Neg Bw6 | Neg | Neg | +++ |
| 39 | NAD | Het 50% | NA | NA |
| 42 | NAD | P | Neg | NA |
| 43 | NAD | Neg | 1, 2, 3 | NA |
| 44 | Neg A3, Neg B7, Het Bw4 | Neg | 1, 2, 3 | NA |
| 46 | NAD | P | Neg | NA |
| 47 | NAD | P | NA | NA |
| 48 | Het Bw6 | Het 50% | Neg | NA |
| 49 | Neg A2 | P | 4 | NA |
| 53 | NAD | P | NA | NA |

NAD, no abnormality detected; Neg, negative tumour; Het, heterogeneous tumour expression; P, positive tumour expression; NA, information not available. For p53 staining: +++, strong diffuse nuclear stain in tumour cells; ++, intermediate stain; +, weak but diffuse tumour nuclear stain; ± weak, patchy tumour stain; Neg, negative tumour nuclear stain, similar to negative control.

the TAP-1 and TAP-2 transporter proteins associated with HLA class I antigen processing were found. Heterogeneous expression of TAP-1 protein, but not of TAP-2, was detected in only 2 out of 34 (6%) of the cases. It was also noticed, however, that in these cases defective expression of the HLA-A2 allele was also found.

### ICAM-1 and HLA-DR expression in HNSCC

ICAM-1 expression on tumour cells was lost in 5 out of the 34 (15%) cases examined. A further 7 out of 34 (21%) specimens had heterogeneous expression of this molecule on their tumour cells. Examples of positive and negative tumour staining for ICAM-1 are shown in Figure 2. De novo expression of HLA class II molecules by HNSCC cells, determined by the use of an anti-HLA-DR antibody, was found in 13 out of 34 (38%) specimens. Of these cases, 4 out of 13 (31%) had heterogeneous expression of HLA-DR, while the remaining 9 out of 13 (69%) were considered to be highly positive. Examples of absent and de novo HLA-DR antigen expression in HNSCC specimens are shown in Figure 2.

### Correlations between HLA and ICAM-1 expression with clinicopathological parameters

HLA class I loss, loss of ICAM-1 expression and de novo expression of HLA-DR was found to occur at all sites from which tumour specimens were obtained. No correlation could be found between grade and stage of HNSCC with loss of HLA class I and ICAM-1 expression, but this may be due to the skewed distribution of the specimens. Decreased expression of these molecules occurred in early as well as late stage lesions, which is highlighted by the finding of HLA class I allelic loss in one borderline dysplasia/carcinoma specimen. However, expression of HLA-DR may be associated with well-differentiated tumours other than with moderately/poorly differentiated tumours.

*© Cancer Research Campaign 1997*

**Figure 2** (**A**) Negative expression of HLA-DR by carcinoma cells, in contrast to (**B**) showing positive de novo expression. (**C**) Positive expression of ICAM-1 by both carcinoma and stromal cells, with (**D**) showing loss of this adhesion molecule by tumour cells in another HNSCC specimen (magnification × 340)

No correlations could be made between HLA and ICAM-1 expression with local lymph node involvement.

### Expression of *MAGE* genes and p53 in HNSCC specimens

The expression of the *MAGE-1*, *-2*, *-3*, *-4* genes was determined by reverse transcription PCR in 27 out of 34 HNSCC specimens. Fifteen out of 27 (55.6%) of these tumour samples expressed at least one of these genes. Expression of *MAGE-1* was found in 9 out of 15 (60%), *MAGE-2* in 6 out of 15 (40%), *MAGE-3* in 6 out of 15 (40%) and *MAGE-4* in 9 out of 15 (60%) tumour specimens that were positive for *MAGE*.

Positive nuclear staining for overexpressed p53 protein was detected by immunohistochemistry in 15 out of 21 (71%) specimens.

The expression of these tumour-associated antigens in relation to loss of HLA class I alleles or de novo HLA-DR expression is given in Table 5. Ten out of 15 (66%) tumours expressing *MAGE* genes showed aberrant HLA expression. Eleven out of 15 (73%) tumours showing strong staining for p53 demonstrated altered expression of HLA class I expression compared with two out of six (33%) cases in which p53 overexpression was not detected.

### Discussion

With the use of allele-specific antibodies, this study of HLA class I antigen expression in HNSCC has found defects in allelic expression in 17 out of 34 (50%) of the specimens stained. However, in only 2 out of 34 (6%) cases was heterogeneous expression of HLA class I detected when using the anti-HLA class I antibody W6/32.

Although a lower frequency of loss of W6/32 positivity was found in this study compared with previous studies on HNSCC and HLA class I expression (Esteban et al, 1989; Houck et al, 1990; Mattijsen et al, 1991), which may be accounted for by differences in the tumour samples used, the high frequency of aberrant allelic expression detected implies that previous studies on HLA class I expression using antibodies directed against monomorphic determinants of these molecules may be underestimates. Using an even wider panel of antibodies against these allele-specific determinants, greater amounts of aberrant expression may be detected.

Concordant expression of HLA class I and $\beta_2$m was observed in all but two cases that showed heterogeneous $\beta_2$m and no loss of reactivity with W6/32. Uncoordinated expression of $\beta_2$m and heavy chains has also been observed in lung, colon and cervical cancers (Momburg et al, 1989; Keating et al, 1995; Korkolopoulou et al, 1996).

To account for the discrepancy between the expression of the monomorphic 'framework' determinants of HLA class I molecules, compared with the loss of specific HLA class I alleles, it has been suggested that malignant cells may compensate for the loss of one class I allospecificity by overexpressing the remaining ones (Ferrone and Marincola, 1995). This may also help to account for the differences in the expression of certain alleles on HNSCC cells, with some alleles (HLA-B7, HLA-A3) being down-regulated to a much greater degree than others (HLA-B8, HLA-A1) on the surface of tumour cells.

In cervical cancer, tumour cells that have decreased expression of the HLA-B7 allele have been associated with a high propensity for lymph node metastases and a poorer clinical outcome (Honma et al, 1994; Ellis et al, 1995). It is thought that the expression of this allele may be important in the control of HPV-associated carcinoma, by playing a dominant role in the presentation of immunogenic HPV-derived peptides to cytotoxic T-cells (Ellis et al, 1995; Keating et al, 1995). By decreasing the expression of this allele, and possibly overexpressing other HLA class I alleles, then tumour cells can decrease the density of immunogenic peptide–MHC complexes on their cell surface to avoid recognition and stimulation of anti-tumour CTL responses (Rivoltini et al, 1995).

As the epithelial cells of the human upper aerodigestive tract resemble those of the female cervical cavity, these cells are also susceptible to HPV infection, with HPV proteins being detected in 30–40% of oral cancer specimens (Gujuluva et al, 1996). Cytotoxic T-lymphocytes have also been isolated in vitro from tumour-infiltrating lymphocytes within HNSCC biopsies, and they demonstrate MHC class I restricted killing of HPV-infected SCC cells (Hald et al, 1995). Whether the loss of the HLA-B7 allele in HNSCC has prognostic implications similar to that in cervical cancer has still to be determined. Because of the limited sample size and follow-up data, no conclusions can be made from this study. However, the greater amounts of loss of HLA class I alleles in two out of four of the lymph node metastases compared with their associated primary tumours suggests that the loss of HLA class I may have a role in the progression of HNSCC.

The mechanisms behind down-regulated HLA class I expression on the surface of tumour cells are multiple and include defects in $\beta_2$m, genetic loss of class I $\alpha$-chains, gene rearrangements, altered transcriptional control, oncogenic activation, viral proteins and altered functions of TAP and proteasome subunits involved in the antigen-processing pathway (Ferrone and Marincola 1995; Garrido et al, 1995; Seliger et al, 1996). Although decreased expression of TAP-1 has been found to occur to a significant level in cervical, lung and breast cancer (Cromme et al, 1994; Kaklamanis et al, 1995; Korkolopoulou et al, 1996), heterogeneous expression of TAP-1 was only found in 2 out of 34 HNSCC specimens stained in this study. This high frequency of TAP-1 expression may be related to the high levels of expression of HLA class I molecules detected by W6/32, with the majority of allelic loss being due to other mechanisms. It was noticed however that in the two specimens in which heterogeneous expression of TAP-1 was found, defective expression of the HLA-A2 allele was also present.

The up-regulated expression of HLA-DR (HLA class II) was found in 38 of the tumour specimens stained. Other studies on HLA-DR expression in HNSCC give varying figures, ranging from as low as 8% (Esteban et al, 1989) to as high as 70% (Houck et al, 1990; Mattijssen et al, 1991). Previous reports have also differed as to what type of tumour predominantly express HLA class II molecules, with Esteban et al (1989) correlating HLA-DR expression to well-differentiated tumours but with others stating that class II positivity was associated mainly with poorly differentiated tumours (Houck et al, 1990; Mattijssen et al, 1991). In this study, although no correlations could be made regarding HLA-DR positivity with site, stage and regional lymph node involvement, it was observed that a high frequency of well-differentiated tumours showed de novo expression of HLA-DR compared with moderately/poorly differentiated HNSCC.

The functional significance of HLA class II molecules on HNSCC cells is debatable, with de novo expression of HLA-DR being related to the presence of cytokines (e.g. IFN-$\gamma$, TNF-$\alpha$) released by tumour-infiltrating lymphocytes (Matsushita et al, 1996), the differentiation status of the cell or as a result of oncogenic transformation. Samukawa (1993) has found a significant $CD8^+$ $CD3^+$ T-cell infiltrate in head and neck carcinomas expressing HLA-DR compared with a $CD4^+$ $CD3^+$ infiltrate in HLA-DR-negative tumours. In vitro findings have also suggested that both MHC class I and class II molecules expressed on head and neck tumour cells play critical roles in inducing tumour-specific $CD8^+$ and $CD4^+$ cytotoxic T-lymphocytes (Chikamatsu et al, 1994).

The expression of ICAM-1 was found to be aberrant in a significant proportion of the HNSCC tumour specimens stained. Down-regulation of this intercellular adhesion molecule on the surface of tumour cells makes them less susceptible to lysis by immune effector cells (Vánky et al, 1995), which include not only cytotoxic T-cells but also NK cells, LAK cells and antibody-dependent cytotoxicity mediated by monocytes and granulocytes (Springer, 1990). Hence, decreased ICAM-1 expression may serve as an additional immunological escape mechanism by tumour cells.

Altered ICAM-1 expression may also affect the interaction between tumour cells and the surrounding matrix, as ICAM-1 interacts with fibrinogen and the extracellular matrix factor hyaluronan (van de Stolpe and van der Saag, 1996). By affecting attachment to the surrounding stroma, altered ICAM-1 expression may be an important factor in the development of metastases; although, in this study, no correlation was found between decreased ICAM-1 expression on HNSCC cells in relation to regional lymph node involvement.

For the presentation of tumour antigens to the immune system, the expression of HLA class I molecules by tumour cells, as well as the antigen, is needed. With the isolation of antigenic peptides derived from tumour antigens that bind to HLA class I molecules, clinical trials of peptide-based cancer vaccines have started (Marchand et al, 1995). Although the HLA type of the patient is usually determined from a blood sample, the decreased expression of specific HLA alleles on the tumour cell surface however may mean that the tumour no longer expresses the restricting HLA class I allele. Thus, the patient's tumour cells will no longer present the antigenic peptide against which a peptide vaccine tries to induce an immune response.

Table 5 demonstrates that although a patient's tumour may express the tumour-associated antigens p53 and *MAGE* genes, HLA class I allele expression can frequently be deviant. In the case of p53 overexpression, over 70% of cases harboured HLA aberration. Both p53- and *MAGE* gene-encoded peptides are potential targets for immune attack (Wiedenfeld et al, 1994; Boon et al, 1995) and loss of presenting HLA molecules may influence processing of immunogenic peptides and consequent immune recognition. Hence, HLA typing of the tumour itself, as well as the patient's blood, may be needed to determine whether the necessary HLA alleles are expressed. Also, a multi-peptide vaccine strategy using different HLA class I alleles binding different antigenic peptides needs to be sought to avoid the 'immunoselection' of these antigen-loss variants (Lehmann et al, 1995; Maeurer et al, 1996). Other methods of vaccination must also be devised that can effectively trigger an anti-tumour response. For example, genetically modified bacteria or antigen-presenting cells, presenting a wide array of immunogenic peptide–MHC complexes and co-stimulatory molecules on their cell

© Cancer Research Campaign 1997

surface, as well as secreting cytokines locally at the tumour site, may produce a long-lived, multi-pronged CTL attack (Irvine and Restifo, 1995).

The role of cytokines, such as TNF-α and IFN-γ, may also be useful in the adjuvant setting because of their ability to up-regulate MHC class I and ICAM-1 and also to induce MHC class II expression (Farrar and Schreiber, 1993; Scher et al, 1993; Ishii et al, 1994), especially if these molecules are associated with the production of anti-tumour CTLs and a better prognosis for head and neck cancer patients.

### ACKNOWLEDGEMENT

This research work was supported by a grant from the Yorkshire Cancer Research Campaign.

### REFERENCES

Boon T, Gajewski F and Coulie PG (1995) From defined human tumour antigens to effective immunisation? *Immunol Today* **16**: 334–335

Chikamatsu K, Eura M, Matsuoka H, Nurakami H and Fukiage T (1994) The role of major histocompatibility complex expression on head and neck cancer cells in the induction of autologous cytotoxic T-lymphocytes. *Cancer Immunol Immunother* **38**: 358–364

Cromme FV, Airey J, Heemels M-T, Ploegh HL, Keating PJ, Stern PL, Meijer CJLM and Walboomers JMM (1994) Loss of transporter protein, encoded by TAP-1 gene, is highly correlated with loss of HLA expression in cervical carcinomas. *J Exp Med* **179**: 335–340

Ellis JRM, Keating PJ, Baird J, Hounsell EF, Renouf DV, Rowe M, Hopkins D, Duggan-Keen MF, Bartholomew JS, Young LS and Stern PL (1995) The association of an HPV 16 oncogene variant with HLA-B7 has implications for vaccine design in cervical cancer. *Nature Med* **1**: 464–470

Esteban F, Concha A, Huelin C, Pérez-Ayala M, Pedrinaci S, Ruiz-Cabbello F and Garrido F (1989) Histocompatibility antigens in primary and metastatic squamous cell carcinoma of the larynx. *Int J Cancer* **43**: 436–442

Farrar MA and Schreiber RD (1993) The molecular cell biology of interferon γ and its receptor. Annu Rev Immunol **11**: 571–611

Ferrone S and Marincola FM (1995) Loss of HLA class I antigens by melanoma cells: molecular mechanisms, functional significance and clinical relevance. *Immunol Today* **16**: 487–494

Garrido F, Cabrera T, Concha A, Glew S, Ruiz-Cabello F and Stern PL (1993) Natural history of HLA expression during tumour development. *Immunol Today* **14**: 491–499

Garrido F, Cabrera T, Lopez-Nevot MA and Ruiz-Carbello F (1995) HLA class I antigens in human tumors. *Adv Cancer Res* **67**: 155–195

Graubert TA and Ley TJ (1996) How do lymphocytes kill tumour cells? *Clin Cancer Res* **2**: 785–789

Gujuluva C, Shin K-H and Park N-H (1996) Role of HPV in tumorigenesis of oral keratinocytes: Implication of p53, p21 $^{WAF1/cip1}$, gadd 45, cyclins, cyclin-dependent kinases and PCNA in oral cancer. *Int J Oncol* **8**: 21–28

Hald J, Rasmussen N and Claesson MH (1995) Tumour-infiltrating lymphocytes mediate lysis of autologous squamous cell carcinomas of the head and neck. *Cancer Immunol Immunother* **39**: 383–390

Honma S, Tsukada S, Honda S, Nakamura M, Takakuma K, Maruhashi T, Kodama S, Kanazawa K, Takahashi T and Tanaka T (1994) Biological–clinical significance of selective loss of HLA-class I allelic product expression in squamous cell carcinoma of the uterine cervix. *Int J Cancer* **57**: 650–655

Houck JR, Sexton M and Zajdel G (1990) HLA class I and class II antigen expression on squamous cell carcinoma of the head and neck. *Arch Otolaryngol Head Neck Surg* **116**: 1181–1185

Ioachim H (1990) The opportunistic tumours of immune deficiency. *Adv Cancer Res* **54**: 301–317

Irvine KR and Restifo NP (1995) The next wave of recombinant and synthetic anti-cancer vaccines. *Semin Cancer Biol* **6**: 337–347

Ishii H, Gochi A and Orita K (1994) Enhancement of cell surface ICAM-1 and HLA class I antigens in human gastric cancer cell lines by IFN-γ. *Acta Med Okayama* **48**: 73–79

Janeway Jr. CA and Travers P (1994) *Immunobiology: the Immune System in Health and Disease.* Current Biology: London

June CH, Bluestone JA, Nadler LM and Thompson CB (1994) The B7 and CD28 receptor families. *Immunol Today* **15**: 321–331

Kaklamanis L, Leek R, Koukourakis M, Gatter KC and Harris AL (1995) Loss of transporter in antigen processing 1 transport protein and major histocompatibility complex class I molecules in metastatic versus primary breast cancer. *Cancer Res* **55**: 5191–5194

Keating PJ, Cromme FV, Duggan-Keen M, Snijders PJF, Walboomers JMM, Hunter RD, Dyer PA and Stern PL (1995) Frequency of down-regulation of individual HLA-A and -B alleles in cervical carcinomas in relation to TAP-1 expression. *Br J Cancer* **72**: 405–411

Korkolopoulou P, Kaklamanis L, Pezzella F, Harris AL and Gatter KC (1996) Loss of antigen-presenting molecules (MHC class I and TAP-1) in lung cancer. *Br J Cancer* **73**: 148–153

Lehmann F, Marchand M, Hainaut P, Pouillart P, Sastre X, Ikeda H, Boon T and Coulie PG (1995) Differences in the antigens recognised by cytolytic T cells on two successive metastases of a melanoma patient are consistent with immune selection. *Eur J Immunol* **25**: 340–347

Maeurer MJ, Gollin SM, Storkus WJ, Swaney W, Karbach J, Martin D, Castelli C, Salter R, Knuth A and Lotze MJ (1996) Tumour escape from immune recognition: Loss of HLA-A2 melanoma cell surface expression is associated with a complex rearrangement of the short arm of chromosome 6. *Clin Cancer Res* **2**: 641–652

Marchand M, Weynants P, Rankin E, Arienti F, Belli F, Parmiani G, Cascinelli N, Bourlond A, Vanwijck R, Humblet Y, Canon J-L, Laurent C, Naeyaert J-M, Plagne R, Deraemaeker R, Knuth A, Jäger E, Brasseur F, Hermann J, Coulie PG and Boon T (1995) Tumour regression responses in melanoma patients treated with a peptide encoded by gene MAGE-3. *Int J Cancer* **63**: 883–885

Matsushita K, Takenouchi T, Kobayashi S, Hayashi H, Okuyama K, Ochiai T, Mikata A and Isono K (1996) HLA-DR antigen expression on colorectal carcinomas: influence of expression by IFNγ *in situ*, and its association with tumour progression. *Br J Cancer* **73**: 644–648

Mattijsen V, De Mulder PHM, Schalkwijk L, Manni JJ, Van't Hof-Grootenboer B and Ruiter DJ (1991) HLA antigen expression in routinely processed head and neck squamous cell carcinoma primary lesions of different sites. *Int J Cancer* **Suppl. 6**: 95–100

Momburg F, Ziegler A, Harpprecht J, Moller P, Moldenhaur G and Hammerling GT (1989) Selective loss of HLA-A or HLA-B antigen expression in colon carcinoma. *J Immunol* **142**: 352–358

Mulcahy KA, Rimoldi D, Brasseur F, Rodgers S, Lienard D, Marchand M, Rennie IG, Murray AK, McIntyre CA, Platts KE, Leyvraz S, Boon T and Rees RC (1996) Infrequent expression of the MAGE gene family in uveal melanomas. *Int J Cancer* **66**: 738–742

Neefjes JJ and Ploegh HL (1992) Intracellular transport of MHC class II molecules. *Immunol Today* **13**: 179–183

Pardoll DM (1993) Cancer vaccines. *Immunol Today* **14**: 310–316

Parham P and Ohta T (1996) Population biology of antigen presentation by MHC class I molecules. *Science* **272**: 67–74

Restifo NP and Wunderlich JR (1995) Biology of cellular immune responses. In *Biologic Therapy of Cancer*, 2nd edn, De Vita Jr. VT, Hellman S and Rosenberg SA. (eds), pp. 3–37. JB Lippincott: Philadelphia

Rivoltini L, Barracchin KC, Viggiano V, Kawakami Y, Smith A, Mixon A, Restifo NP, Topalian SL, Simonis TB, Rosenberg SA and Marincola FM (1995) Quantitative correlation between HLA class I allele expression and recognition of melanoma cells by antigen-specific cytotoxic T-lymphocytes. *Cancer Res* **55**: 3149–3157

Samukawa T (1993) Expression of HLA-DR antigen on head and neck carcinomas – immunohistological study (Japanese). *Nippon-Jibiinkoka-Gattai-Kaiho* **96**: 88–97

Scher RL, Koch WM and Richtsmeier WJ (1993) Induction of the intercellular adhesion molecule (ICAM-1) on squamous cell carcinoma by interferon gamma. *Arch Otolaryngol Head Neck Surg* **119**: 432–438

Seliger B, Höhne A, Knuth A, Bernhard H, Meyer T, Tampe R, Momburg F and Huber C (1996) Analysis of the major histocompatibility class I antigen presentation machinery in normal and malignant renal cells: evidence for deficiencies associated with transformation and progression. *Cancer Res* **56**: 1756–1760

Springer TA (1990) Adhesion receptors of the immune system. *Nature* **346**: 425–433

Van de Stolpe A and Van der Saag PT (1996) Intercellular adhesion molecule-1. *J Mol Med* **74**: 13–33

Vánky F, Hising C, Sjöwall K, Larsson B, Rodriguez L, Orre L and Klein E (1995) Immunogeneity and immunosensitivity of ex vivo human carcinomas: interferon γ and tumour necrosis factor α treatment of tumour cells potentiates their interaction with autologous blood lymphocytes. *Cancer Immunol Immunother* **41**: 217–226

© Cancer Research Campaign 1997

Wiedenfeld EA, Fernandez-Vina M, Berzofsky JA and Carbone DP (1994) Evidence for selection against human lung cancers bearing p53 missense mutations which occur within the HLA A*0201 peptide consensus motif. *Cancer Res* **94**: 1175–1177

York IA and Rock KL (1996) Antigen processing and presentation by the class I major histocompatibility complex. *Annu Rev Immunol* **14**: 369–396

*© Cancer Research Campaign 1997*